# The epidemiology of venous thromboembolism

**DOI:** 10.1007/s11239-015-1311-6

**Published:** 2016-01-16

**Authors:** John A. Heit, Frederick A. Spencer, Richard H. White

**Affiliations:** Division of Cardiovascular Diseases (JAH), Mayo Clinic, Hematology Research-Stabile 660, 200 First Street SW, Rochester, MN 55905 USA; Division of Cardiology (FAS), McMaster University, Hamilton, ON USA; Division of General Internal Medicine (RHW), University of California, Davis, Sacramento, CA USA

**Keywords:** Venous thromboembolism, Pulmonary embolism, Deep vein thrombosis, Thrombophlebitis, Epidemiology

## Abstract

Venous thromboembolism (VTE) is categorized by the U.S. Surgeon General as a major public health problem. VTE is relatively common and associated with reduced survival and substantial health-care costs, and recurs frequently. VTE is a complex (multifactorial) disease, involving interactions between acquired or inherited predispositions to thrombosis and VTE risk factors, including increasing patient age and obesity, hospitalization for surgery or acute illness, nursing-home confinement, active cancer, trauma or fracture, immobility or leg paresis, superficial vein thrombosis, and, in women, pregnancy and puerperium, oral contraception, and hormone therapy. Although independent VTE risk factors and predictors of VTE recurrence have been identified, and effective primary and secondary prophylaxis is available, the occurrence of VTE seems to be relatively constant, or even increasing.

## Introduction

Thrombosis can affect virtually any venous circulation. This chapter focuses on the epidemiology of venous thromboembolism (VTE), including deep vein thrombosis (DVT) of the leg or pelvis, and its complication, pulmonary embolism (PE). Thrombosis affecting the superficial leg (e.g. saphenous) veins and other venous circulations (e.g. arm, cerebral, mesenteric, renal, hepatic, portal veins) is beyond the scope of this chapter. VTE is a complex (multifactorial) disease, involving interaction between acquired or inherited predispositions to thrombosis (i.e. thrombophilia) and environmental exposures (i.e. clinical risk factors) [1–5]. Moreover, the type of VTE event (PE vs. DVT) may also be partly heritable [6, 7]. Most studies of VTE epidemiology addressed populations of predominantly European origin, and the data discussed in this chapter mainly relate to these populations. Where available, data from populations originating from other continents are presented.

## Methods

This chapter focuses on population-based studies and addresses nine questions regarding the epidemiology of VTE (Table 1). Questions were developed by consensus from the authors. The literature addressing the above questions was reviewed by searching electronic databases (PubMed, Medline), including the references of all identified papers and the author’s personal libraries. Since this review is informational only and does not address diagnosis, treatment or management of VTE, there are no associated guidance statements. The post thrombotic syndrome and the epidemiology of inherited and acquired thrombophilia are reviewed in Chapters 9 and 10, respectively.

**Table 1:** Guidance questions to be considered

(1) What is the incidence of VTE, PE with or without (±) DVT and leg DVT alone, both overall, and by age, sex and race, and by idiopathic versus secondary VTE?
(2) What are the trends in incidence over time of overall VTE, leg DVT alone and PE ± DVT, and of idiopathic versus secondary VTE? How are these trends affected by changes in diagnostic test utilization, imaging resolution and autopsy rates over time?
(3) What is the cumulative incidence of VTE recurrence, both overall and by leg DVT alone versus PE ± DVT?
(4) What baseline and time-dependent characteristics are independent predictors of VTE recurrence after adjustment for primary treatment and secondary prophylaxis? Within the major predictors of VTE recurrence, can the individual patient be further stratified into high and low risk? How well do available VTE recurrence risk-prediction scores operate in predicting recurrence?
(5) What are the attack rates (i.e. incident and recurrent VTE) of VTE, both overall and by hospitalization-related versus community-acquired VTE? What is the total number of VTE events (incident and recurrent) per year in the USA?
(6) What are the VTE-attributable costs?
(7) What is the survival after VTE overall, and after leg DVT alone vs. PE ± DVT. What are the independent predictors of survival? What are the trends in survival over time after PE ± DVT?
(8) What are the independent risk factors for VTE? How well do incident VTE risk prediction scores operate in predicting incident VTE for the individual?

## Questions

1. What is the incidence of VTE, PE with or without (±) DVT and leg DVT alone, both overall, and by age, sex and race, and by idiopathic versus secondary VTE?

The estimated average annual incidence rate of overall VTE among persons of European ancestry ranges from 104 to 183 per 100,000 person-years [8–18]; overall VTE incidence is similar to that of stroke [19, 20]. Overall VTE incidence may be higher in African-Americans [21–23] and lower in Asians [24], and Asian- [25, 26] and Native-Americans [27], and may differ among African-Americans by United States region [23]. Reported incidence rates for PE ± DVT, and for leg DVT alone, range from 29 to 78, and 45 to 117, per 100,000 person-years, respectively [10, 12, 14–18].

**Fig. 1:**
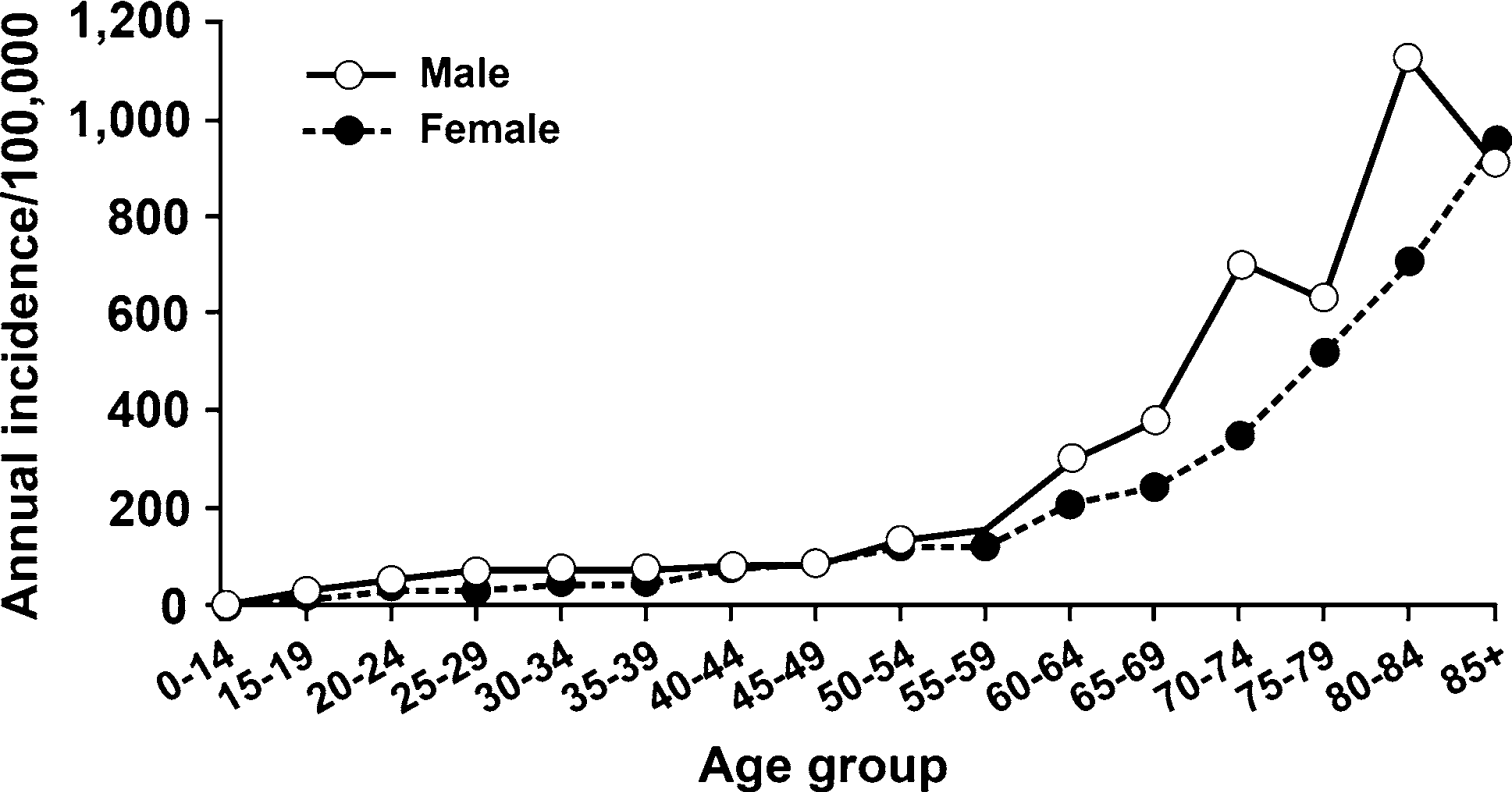
Annual incidence of venous thromboembolism by age and sex [10]

**Fig. 2:**
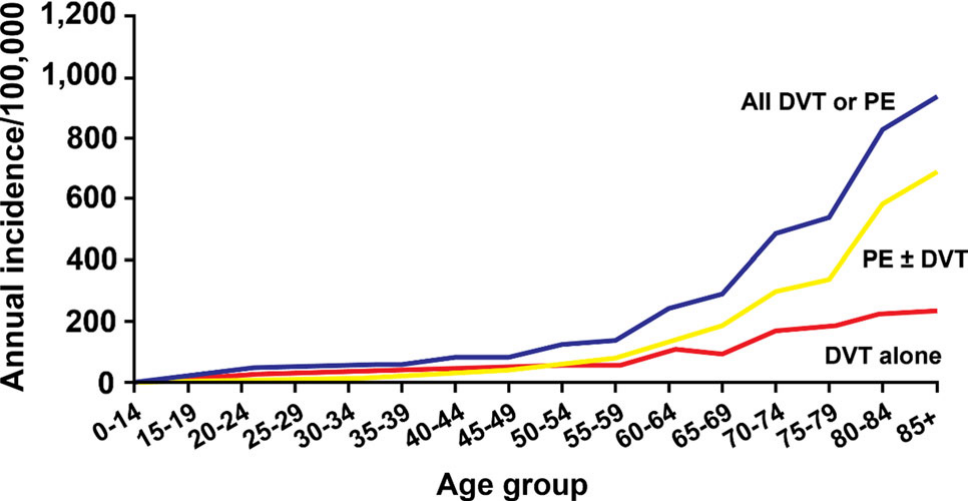
Annual incidence of all venous thromboembolism, deep vein thrombosis (DVT) alone, and pulmonary embolism with or without deep vein thrombosis (PE ± DVT) by age [10]

VTE is predominantly a disease of older age; VTE is rare prior to late adolescence [8, 10–15, 18]. Incidence rates increase markedly with age for both men and women (Fig. 1) and for both DVT and PE (Fig. 2) [10, 14, 15]. The overall age-adjusted incidence rate is higher for men (130 per 100,000) then women (110 per 100,000; male:female sex ratio is 1.2:1) [10, 15]. Incidence rates are somewhat higher in women during childbearing years, while incidence rates after age 45 years are generally higher in men. PE accounts for an increasing proportion of VTE with increasing age for both sexes [10]. The percentage of incident VTE events that are idiopathic ranges from 25 to 40 % [26, 28, 29]. In one study, 19 % of events among Asian/Pacific Islanders were idiopathic [26].

(2) What are the trends in incidence over time of overall VTE, leg DVT alone and PE ± DVT, and of idiopathic versus secondary VTE? How are these trends affected by changes in diagnostic test utilization, imaging resolution and autopsy rates over time?

**Fig. 3:**
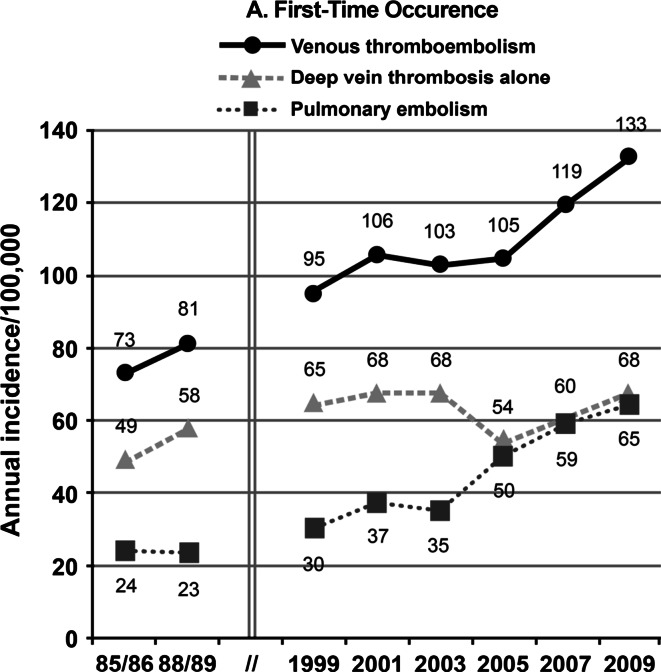
Secular trends in the incidence of venous thromboembolism, deep vein thrombosis alone, and pulmonary embolism [18]

Data on trends in VTE incidence are limited; overall VTE incidence rates as well as incidence rates for PE ± DVT and DVT alone either remained relatively constant or increased for the period, 1981-2000, with a significant increase in the overall VTE incidence rate from 2001 to 2009, mostly due to an increasing incidence of PE ± DVT (Fig. 3) [10, 14, 18, 30]. The incidence rates of incident cancer-associated VTE, secondary non cancer-associated VTE and idiopathic VTE, 1999–2009, appear to be relatively constant [29]. The observed increase in overall VTE and PE ± DVT incidence rates over the most recent time period may, in part, reflect increased utilization of objective imaging and improved image resolution, particularly computed tomography, pulmonary angiography, and magnetic resonance imaging [18].

(3) What is the cumulative incidence of VTE recurrence, both overall and by leg DVT alone vs. PE ± DVT?

**Fig. 4:**
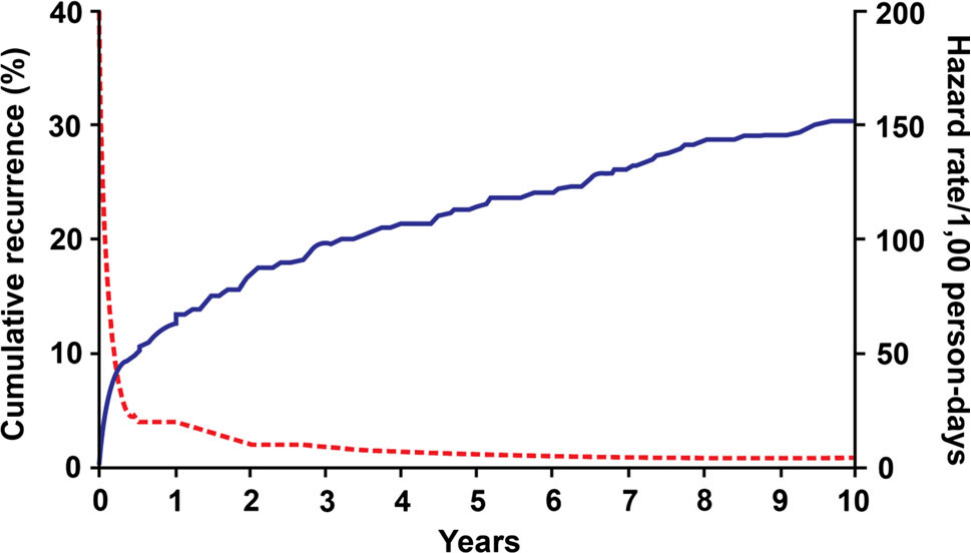
Cumulative incidence of first venous thromboembolism recurrence (*continuous line*), and the hazard of first recurrence per 1000 person-days (*dotted line*) [32]

VTE recurs frequently; about 30 % of patients develop recurrence within the next 10 years (Fig. 4) [16, 31–41]; reported incidence rates of recurrent overall VTE, PE ± DVT and DVT alone range from 19 to 39, 4-13, and 15 to 29 per 100,000 person-years, respectively [18]. The hazard of recurrence varies with the time since the incident event and is highest within the first 6–12 months but never falls to zero. While secondary prophylaxis is effective in preventing recurrence, the duration of acute treatment does not affect the rate of recurrence after three months of adequate anticoagulation has been completed [34, 35, 42–46]. These data suggest that VTE is a chronic disease with episodic recurrence [32, 33, 47, 48].

(4) What baseline and time-dependent characteristics are independent predictors of VTE recurrence after adjustment for primary treatment and secondary prophylaxis? Within the major predictors of VTE recurrence, can the individual patient be further stratified into high and low risk? How well do available VTE recurrence risk-prediction scores operate in predicting recurrence?

Independent predictors of recurrence include increasing patient age (per 10 years [32, 33, 35, 36, 49–54] and body mass index (per 10 kg/m^2^) [32, 52, 54–57] male sex [32, 35, 51, 57–65], active cancer [12, 31, 32, 49, 66–71], and neurologic disease with leg paresis [32]. Additional predictors of recurrence include idiopathic VTE [31, 36, 44, 59, 67, 68, 72–74], a lupus anticoagulant or antiphospholipid antibody [43, 75], antithrombin, protein C or protein S deficiency [76–78], hyperhomocysteinemia [79], a persistently increased plasma D-dimer among patients with idiopathic VTE [80–83], and possibly residual vein thrombosis [84, 85]. Among active cancer patients, VTE recurrence risk can be further stratified by cancer site (pancreatic, brain, lung and ovarian cancer, myeloproliferative or myelodysplastic disorders), stage IV cancer, cancer stage progression and leg paresis [71].

Of equal importance, several risk factors present at the time of the incident VTE event are associated with either a reduced risk of recurrence or are not predictive of recurrence [31–33, 45, 59, 86]. For women, pregnancy or the postpartum state [32, 39], oral contraceptive use [32], hormone therapy [63, 87], and gynecologic surgery [32] are associated with a reduced risk of recurrence. Recent surgery and trauma/fracture either have no effect [32] or predict a reduced risk of recurrence [31, 88]. Additional characteristics having no significant effect on recurrence risk include recent immobilization, tamoxifen therapy, and failed prophylaxis [32]. For these patients, and for patients with isolated calf vein thrombosis, a shorter duration (i.e., 6 weeks) of acute therapy likely is adequate [45, 67]. While data on the type of incident event type (DVT alone vs. PE ± DVT) as a predictor of recurrence are conflicting [32, 36, 37, 45, 50, 57, 89–92], patients with a recurrent event are significantly more likely to have the same type of VTE event as the incident event type [50].

Several VTE recurrence prediction scores have been derived for stratifying recurrence risk among patients with incident idiopathic or cancer-associated VTE. In the “Men continue and HERDOO2″ score, there were no predictors of a reduced risk of recurrence among men with incident idiopathic VTE. In contrast, women with idiopathic VTE who had ≤1 of the following risk factors had a significantly lower risk of VTE recurrence: (1) older age (≥ 65 years), (2) obesity (BMI ≥ 30 kg/m^2^), (3) an increased D-dimer prior to stopping warfarin therapy and (4) signs of post thrombotic syndrome [93]. In the Vienna prediction model, male sex, incident VTE site (PE and proximal DVT vs. isolated calf DVT) and increasing D-dimer level were predictors of recurrence after idiopathic incident VTE [94]. In the DASH prediction score, a persistently increased D-dimer after stopping anticoagulation therapy, age <50 years, male sex and VTE unrelated to hormonal therapy (in women) predicted an increased risk of recurrence after an “idiopathic” incident VTE [95]. Thus, the only inconsistent risk factor in these models is the effect of patient age, with older and younger age being associated with a higher recurrence risk among women in the HERDOO2 model and among men and women in the DASH model, respectively [93, 95], while patient age was not a predictor of recurrence in the Vienna model [94]. Depending on the model, patients with a low score had 1.6–4.4 % per year recurrence rates [96, 97]. Assuming these recurrence rates are acceptable, about 50 % of patients with idiopathic incident VTE and a low prediction score could avoid secondary prophylaxis [93–95]. Among active cancer patients with VTE, patient sex, cancer site (lung, breast), cancer stage and prior VTE were predictors of VTE recurrence while on anticoagulation therapy [70, 98].

(5) What are the attack rates (i.e. incident and recurrent VTE) of VTE, both overall and by hospitalization-related versus community-acquired VTE? What is the total number of VTE events (incident and recurrent) per year in the USA?

Estimated overall VTE attack rates range from 142 to over 300 per 100,000 person-years; estimated attack rates for PE ± DVT and DVT alone range from 51 to 75 and 91 to 255 per 100,000, respectively [18, 99]. Overall VTE attack rates related to current or recent hospitalization are much higher compared to rates among persons residing in the community (330 vs. 8 per 100,000, respectively) [99].

There are few data on the total number of VTE events (incident and recurrent) occurring in the USA per year, and available estimates vary widely. Using age- and sex-specific incidence rates for the five-year time period, 1991–95, projected to the 2000 United States white population, at least 260,000 first-lifetime cases of VTE occur among whites in the United States annually [13]. If incidence rates among African-Americans are similar, then 27,000 additional incident cases occur among African-Americans in the United States annually. In an incidence-based modeling study that included both hospital- and community-acquired, incident and recurrent VTE events, an estimated 600,000 non-fatal VTE events (370,000 DVT and 270,000 PE) occurred in the USA in 2005; of the total, approximately two-thirds were related to current or recent hospitalization [13]. Using 2007-2009 National Hospital Discharge Survey discharge diagnosis codes, an estimated average of about 548,000 hospitalizations with VTE occurred each year among USA residents aged ≥18 years, of which 349,000 were DVT and 278,000 were PE [100].

(6) What are the VTE-attributable costs?

In population-based studies, the adjusted mean predicted costs were 2.5-fold higher for patients with VTE related to current or recent hospitalization for acute medical illness ($62,838) compared to hospitalized controls matched on active cancer status ($24,464; p < 0.001) from the VTE event date (or index date for controls) to 5 years post index; cost differences between cases and controls were greatest within the first 3 months (mean difference = $16,897) [101]. Similarly, the adjusted mean predicted costs were 1.5-fold higher for patients with VTE related to current or recent hospitalization for major surgery ($55,956) compared to hospitalized controls matched to cases on type of surgery and active cancer status ($32,718; p < 0.001) from the VTE event date (or index date for controls) to 5 years post index [102]. Cost differences between cases and controls were also greatest within the first 3 months after index (mean difference = $12,381). Costs were significantly higher for cases than controls (mean difference = $10,797) from 3 months to up to 5 years post-index and together accounted for about half of the overall cost difference. Finally, the adjusted mean predicted costs were over 2-fold higher for patients with VTE related to active cancer ($52,422) compared to active cancer controls matched on the duration of active cancer ($23,951; p < 0.001) from the VTE event date (or index date for controls) to 5 years post index [103]. Cost differences between cases and controls were greatest within the first 3 months (mean difference = $16,488) but remained significantly higher for up to 4 years after index.

(7) What is the survival after VTE overall, and after leg DVT alone vs. PE ± DVT. What are the independent predictors of survival? What are the trends in survival over time after PE ± DVT?

Overall, survival after VTE is worse than expected, and survival after PE is much worse than after DVT alone (Table 2) [37, 104–107]. The risk of early death among PE patients is 18-fold higher compared with patients with DVT alone [104]. Pulmonary embolism is an independent predictor of reduced survival for up to 3 months after onset. After 3 months, observed survival after PE is similar to expected survival [15, 104, 105]. For almost one-quarter of PE patients, the initial clinical presentation is sudden death [104]. Independent predictors of reduced early survival after VTE include increasing age, male sex, lower body mass index, confinement to a hospital or nursing home at VTE onset, congestive heart failure, chronic lung disease, serious neurologic disease, and active cancer [41, 104, 105, 108]. Additional clinical predictors of poor early survival after PE include syncope and arterial hypotension [108, 109]. Evidence of right heart failure based on clinical examination, plasma markers (e.g. cardiac troponin T, brain natriuretic peptide) or echocardiography predicts poor survival among normotensive PE patients [108]. Survival over time may be improving for those PE patients living sufficiently long to be diagnosed and treated [104, 106, 110, 111].

**Table 2:** Survival (%) after deep vein thrombosis vs. pulmonary embolism [104]

Time	Deep vein thrombosis alone	Pulmonary embolism
0 days	97.0	76.5
7 days	96.2	71.1
14 days	95.7	68.7
30 days	94.5	66.8
90 days	91.9	62.8
1 year	85.4	57.4
2 years	81.4	53.6
5 years	72.6	47.4
8 years	65.2	41.5

(8) What are the independent risk factors for VTE? How well do incident VTE risk prediction scores operate in predicting incident VTE for the individual?

Independent risk factors for VTE include increasing patient age and body mass index, major surgery, hospitalization for acute medical illness, nursing home confinement, trauma/fracture, active cancer with or without concurrent chemotherapy, central vein catheterization or transvenous pacemaker, prior superficial vein thrombosis, varicose veins, neurologic disease with leg paresis, urinary tract infection, an increased baseline plasma fibrin D-dimer and family history of venous thromboembolism; patients with chronic liver disease have a reduced risk (Table 3) [112–118]. Compared to residents in the community, hospitalized residents have over a 100-fold increased incidence of VTE [119]. Hospitalization and nursing home residence together account for almost 60 % of incident VTE events occurring in the community [28, 120]. Of note, hospitalization for medical illness and hospitalization for surgery account for almost equal proportions of VTE (22 and 24 %, respectively). Nursing home residence independently accounts for over one-tenth of all VTE disease in the community [28, 120].

**Table 3:** Independent risk factors for deep vein thrombosis or pulmonary embolism [117]

Baseline characteristic	Odds ratio	95 % CI
Body mass index (kg/m^2^)	1.08	1.05, 1.11
Major surgery	18.95	9.22, 38.97
Hospitalization for acute medical illness	5.07	3.12, 8.23
Nursing home confinement	4.63	2.77, 7.74
Trauma/fracture	4.56	2.46, 8.46
Active cancer	14.64	7.73, 27.73
Neurologic disease with leg paresis	6.10	1.97, 18.89
Pregnancy or postpartum	4.24	1.30, 13.84
Oral contraceptives	4.03	1.83, 8.89
Estrogen alone	1.81	1.06, 3.09
Non-contraceptive estrogen plus progestin	2.53	1.38, 4.63

VTE risk among surgery patients can be further stratified based on patient age, type of surgery, smoking and the presence of active cancer [121–123]. The incidence of postoperative VTE is increased for surgery patients ≥65 years of age [124]. High VTE-risk surgical procedures include neurosurgery, major orthopedic surgery of the leg, thoracic, abdominal or pelvic surgery for cancer, renal transplantation, and cardiovascular surgery [115, 124, 125]. Obesity [126–129], and poor American Society of Anesthesiology physical status [130] are risk factors for VTE after total hip arthroplasty.

VTE risk among patients hospitalized for acute medical illness may be further stratified based on increasing patient age, obesity, previous VTE, thrombophilia, cancer, recent trauma or surgery, tachycardia, acute myocardial infarction or stroke, leg paresis, congestive heart failure, prolonged immobilization (bed rest), acute infection or rheumatologic disorder, hormone therapy, central venous catheter, admission to an intensive or coronary care unit, white blood cell count and platelet count [131–137].

While risk assessment models for predicting VTE among hospitalized non-surgical patients have been derived, the number, predictor types and strength of association with VTE are highly variable and lack generalizability and adequate validation [138, 139].

Active cancer accounts for almost 20 % of all incident VTE occurring in the community [28, 120]. The risk appears to be higher for patients with cancer of the brain, pancreas, ovary, colon, stomach, lung, kidney and bone [140, 141], and in patients with distant metastases [141]. Cancer patients receiving immunosuppressive or cytotoxic chemotherapy are at even higher risk for VTE [112, 141], including therapy with L-aspariginase [142, 143], thalidomide [144] or lenalidomide [145], or tamoxifen [146]. Routine screening for occult cancer is controversial and likely not warranted. However, if clinical features suggest a possible occult cancer (i.e. idiopathic VTE, especially among patients with abdominal vein or bilateral leg vein thrombosis [147] or in whom VTE recurs [148]) then the only imaging study shown to be useful is a CT scan of the abdomen and pelvis [148]. Among cancer patients, the risk of chemotherapy-associated VTE is increased in patients with pancreatic or gastric cancer, platelet count ≥350 × 10^9^/L, hemoglobin <100 g/L or use of red cell growth factors, leukocyte count ≥11 × 10^9^/L, or body mass index ≥35 kg/m^2^ [149]; biomarkers (plasma soluble P-selectin and D-dimer) add further predictive value [150].

A central venous catheter or transvenous pacemaker accounts for 9 % of all incident VTE occurring in the community [28]. Central venous access via femoral vein catheters is associated with a higher incidence of VTE compared to subclavian vein catheterization [151]. Prior superficial vein thrombosis is an independent risk factor for subsequent DVT or PE remote from the episode of superficial thrombophlebitis [112, 152]. The risk of DVT imparted by varicose veins is uncertain and appears to vary by patient age [112, 153, 154]. Long haul (>4–6 h) air travel is associated with a slightly increased risk for VTE (~1 per 4656 flights [155–157]) that is preventable with elastic stockings [158]. HMG-Coenzyme A reductase inhibitor (statin) therapy may provide a 20–50 % risk reduction for VTE [159–161]. Hypertriglyceridemia doubles the risk of VTE in postmenopausal women [162]. However, the risk associated with atherosclerosis, or other risk factors for atherosclerosis, remains uncertain [118, 163–167]. Diabetes mellitus [117], myocardial infarction [168], current or past tobacco smoking, high-density lipoprotein cholesterol, lipoprotein (a), chronic obstructive pulmonary disease, and renal failure are not independent risk factors for VTE [112, 169, 170]. The risk associated with congestive heart failure, independent of hospitalization, is low [112, 121].

Among women, additional risk factors for VTE include oral contraceptive use [117, 121, 171–173], hormone therapy [117, 174, 175], pregnancy and the postpartum period [121, 172, 176], and therapy with the selective estrogen receptor modulator, raloxifene [177]. First and third generation oral contraceptives convey higher risk than second generation oral contraceptives [173]. Injectable depot-medroxyprogesterone acetate for contraception is associated with a three-fold increased risk for venous thromboembolism, while a levonorgestrel intrauterine device imparts no risk [178]. Hormone therapy is associated with a 2- to fourfold increased risk of VTE [117, 174], but the risk may vary by type of estrogen [179] and there may be no risk with transdermal estrogen therapy [180]. The overall incidence of pregnancy-associated VTE is about 200 per 100,000 woman-years; compared to non-pregnant women of childbearing age, the relative risk is increased about fourfold [176, 181]. The risk during the postpartum period is about fivefold higher than the risk during pregnancy [176]. Prior superficial vein thrombosis is an independent risk factor for VTE during pregnancy or postpartum [182, 183].

Other conditions associated with VTE include autoimmune disorders [167], Behcet’s syndrome, celiac disease [184], heparin-induced thrombocytopenia [185], homocystinuria and hyperhomocysteinemia [186, 187], hyperthyroidism [188], immune thrombocytopenia (ITP) [189, 190], infection [114], inflammatory bowel disease [191], intravascular coagulation and fibrinolysis/disseminated intravascular coagulation (ICF/DIC), myeloproliferative disorders (especially polycythemia rubra vera and essential thrombocythemia) [192, 193], chronic kidney disease with severely reduced glomerular filtration rate [194], nephrotic syndrome [195], paroxysmal nocturnal hemoglobinuria [196], rheumatoid arthritis [197, 198], obstructive sleep apnea [199, 200], thromboangiitis obliterans (Buerger’s disease), thrombotic thrombocytopenic purpura, sickle cell disease [201], systemic lupus erythematosus, and granulomatosis with polyangiitis (Wegener’s) [202].
